# Right ventricle pierced by a traditional sharp hair straightener: a case report

**DOI:** 10.1186/s13019-024-02770-3

**Published:** 2024-06-15

**Authors:** Adama Sawadogo, Gaia Severgnini, Moussa Bazongo, Lassina Konaté, Farid Pingwindé Belem, Youssouf Naré, Alain Sanou, Silvia Perlangeli, Lorena Fénoglio, Alberto Pilozzi, Maurizio Roberto, Marco Zanobini

**Affiliations:** 1 Department of Cardiovascular and Thoracic Surgery, Centre Hospitalier Universitaire de Tengandogo, Ouagadougou, Burkina Faso; 2 Department of Cardiology, Hôpital Princesse Grace, Monaco, Monaco; 3 Department of Cardiovascular Surgery, Centro Cardiologico Monzino IRCCS. Milan, Milano, Italia; 4 Department of Cardiac surgery, Azienda Ospedaliera S.Croce e Carle Cuneo, Cuneo, Italia

**Keywords:** Chest trauma, Cardia foreign body, Right ventricle, Case report

## Abstract

The case presents a traumatic ventricular perforation of a girl, accidentally felt on a sharp instrument. The uniqueness of the case presented is due to the very high infrequency of injuries with this type of sharp object. The 7-year-old girl was transported to the hospital after accidentally falling on a sharp instrument. The child had no signs of heart failure. On opening the chest, it was found that the metal object was lodged in the right ventricle. Quickly proceeded to remove the object and suture the entry hole. After a short hospitalization, the child was discharged completely cured.

## Introduction

**Fig. 1:**
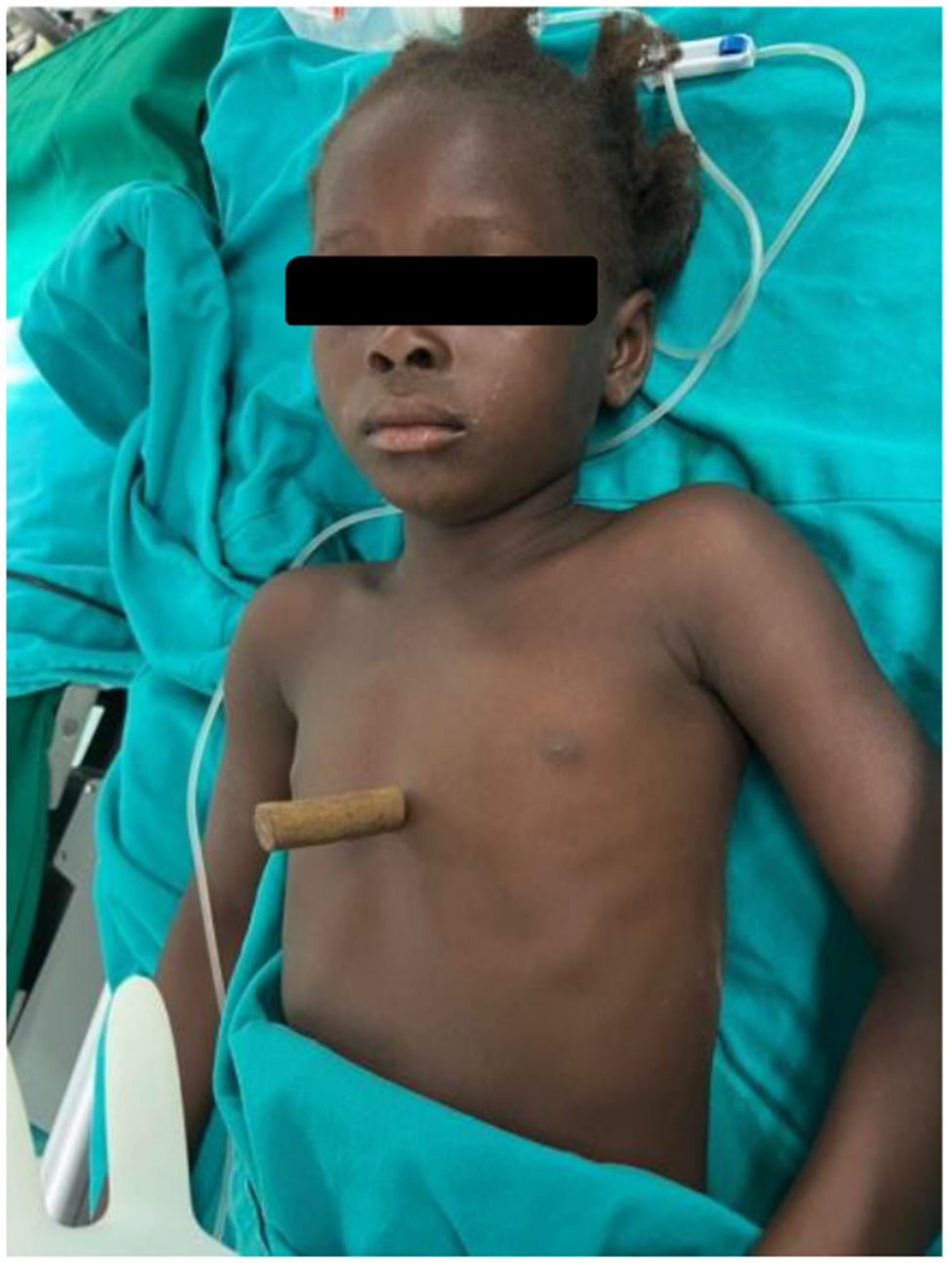
A seven-year-old girl who accidentally fell on a traditional hair straightener

Traumatic ventricular perforation (VP) can occur following chest trauma; some patients, however, may not develop symptoms such as heart failure immediately after injury. Moreover, there is no consensus on the ideal time of surgery once traumatic VP is diagnosed. The treatment strategy can be further complicated when damage to and fracture of other organs necessitate additional surgical interventions. Here, we report a case of traumatic VP caused by blunt chest trauma (Fig. 1) successfully closed via a right ventricular. Our report highlights that traumatic VP should be managed with an optimal surgical strategy according to the patient’s hemodynamics [1]. 

## Case presentation

**Fig. 2:**
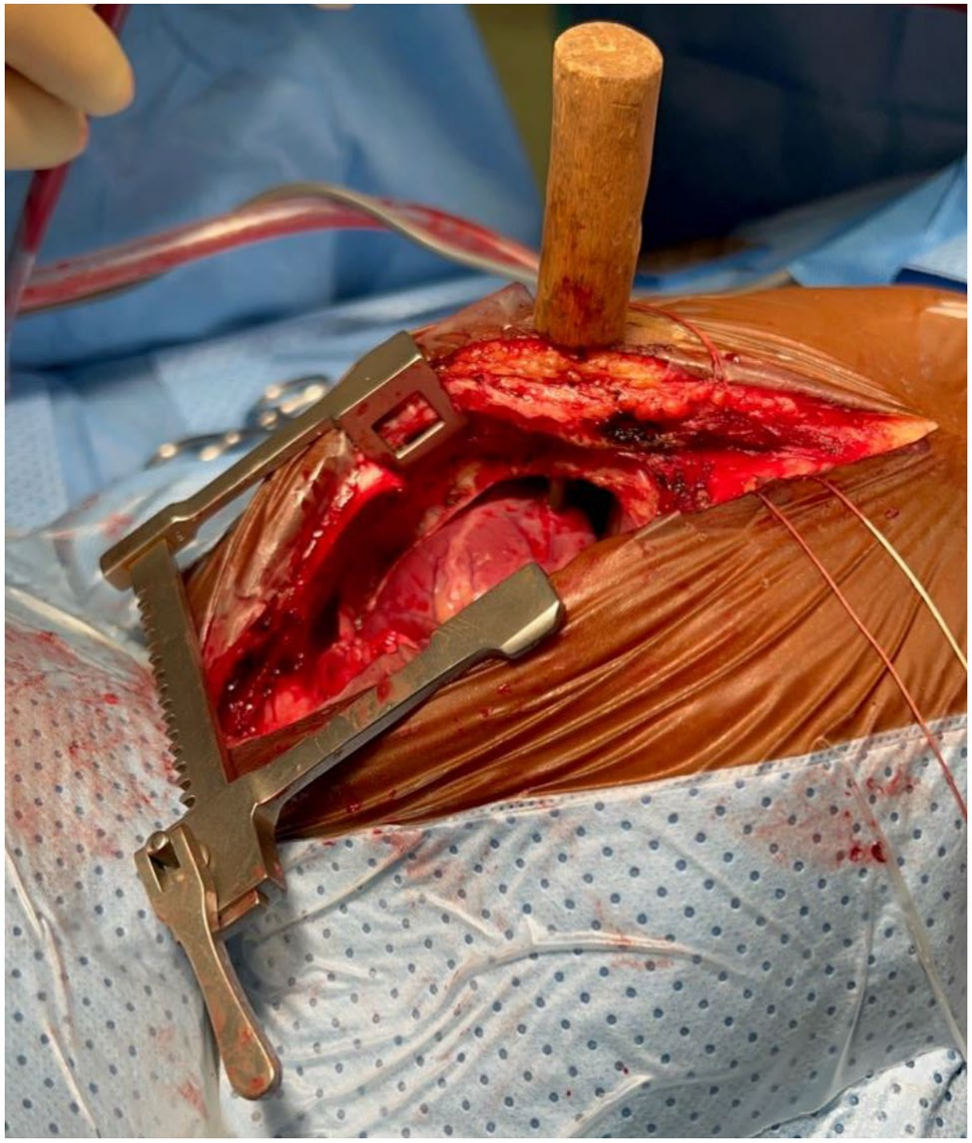
After a sternotomy, the right ventricle wall was pierced by the tool

A 7-year-old girl was transported to Ouagadougu Hospital after accidentally falling on a traditional hair straightener that possesses a sharp, metal part (Fig. 2). This part unfortunately went through the little girl’s chest and pierced her heart in the right ventricle region.

The little girl accompanied by her mother arrived at the hospital after several hours of travel from a small village in Burkina Faso. The instrument was not touched after the accident, and the child arrived in stable hemodynamic condition, with little pain and normal vital parameters. No radiological examinations were performed once she arrived at the hospital facility, because of the urgency of the case and the lack of local availability; the only instrumental examination that could be performed was a transthoracic echocardiogram, which identified the location of the instrument tip at the level of the right ventricle, and allowed us to suppose the non-involvement of structures such as valves or the interventricular septum. No pericardial effusion was shown on examination.

**Fig. 3:**
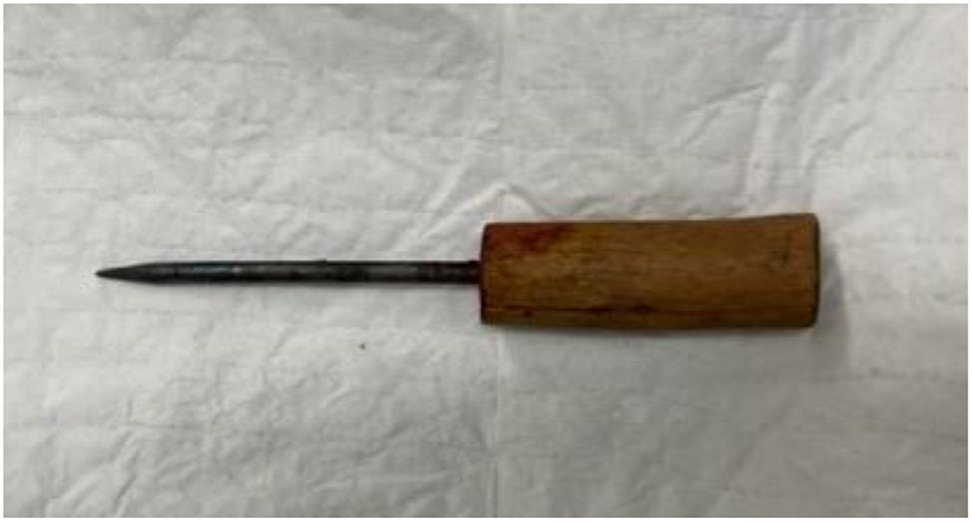
An overview of the traditional hair straightener

Emergency surgery was then performed to remove the instrument. (Fig. 3) Because the instrument was located lateral to the sternum, it was possible to perform a complete median sternotomy with mayo scissors. The sternum was spread just enough to gain access to the right ventricle where the tip of the penetrating instrument could be located. The surgical strategy applied was to proceed with two sutures with pledget in vaycryl 4.0 around the instrument tip; at this point the first and second operators coordinated to perform in a synchronized manner the extraction of the instrument and tighten the stitches with pledget. The stitches were subsequently ligated, and after verification of accurate hemostasis, chest closure could be performed.

The child was transferred to the intensive care unit intubated, under general anaesthesia. The child’s postoperative course was free of major complications. Postoperative recovery was complete, and the child was discharged from the hospital after about 21 days.

## Discussion

It may occasionally happen in emergency rooms that doctors run into an in-situ cardiac foreign body. Symptoms in those cases attributable to these foreign bodies are cardiac tamponade and arrhythmia, that are considered a primary indication for removal. Removal of these objects must be well thought out because it can also lead to further injury or lead to instability for the patient. During the eventual extraction of the object, what the surgeon must be particularly careful about is embolization of material, especially if the bodies is contained within the left heart. What is normally done is manual removal after performing a pledged suture around the foreign body (a double-armed horizontal mattress sutures), then tightened after careful removal of the object [2].

There are studies that have shown that in these cases of cardiac foreign body injury, cardiac tamponade promotes survival precisely because it prevents exsanguination. In contrast, there are studies that show that the protective effect of tamponade is limited and time-dependent [3]. 

Among the earliest cardiac surgeries are those repairing cardiac lacerations by blunt objects, such as the first surgery ever performed by Rehn. The techniques used were initially primordial, in which the piercing instrument was removed and the hole plugged with a finger while suturing the laceration. Important experiences are reported during the war period where thoracic trauma was common [4]. 

The type of access that the surgeon decides to use depends on the location of the injury. In our case, the access that was decided upon was the sternotomy access because the implement lodged in the chest was immediately lateral to the margin of the sternum between the ribs. Other types of accesses may be right or left thoracotomy when the lesions are more lateral to the chest [2]. 

The technique that was decided to use was the one that is used in cases of right ventricle tears due to other causes. The most common causes of perforation of the right ventricle are the placement of devices such as pace makers or ventricular wires. The mechanism of rupture is obviously different, but the suturing techniques can be similar. Even in the case of a post-infarction ventricular tear, the suturing technique is generally a pledget suture. In the presented case of the little girl, the definite advantage was that the tissue on which the suture was placed was healthy, and the instrument entry hole small.

In all cardiac surgery, antibiotic prophylaxis is established to prevent postoperative infections. Several studies [5, 6] show that the most commonly used class of antibiotics are second- or third-generation cephalosporins, and what has emerged and been confirmed in several studies is that the expected duration for a clear reduction in infections is more than 24 h of antibiotic therapy, but not more than 48, as the benefits would not be so evident. Especially in our case, in the days following surgery, the child was treated with empirical antibiotic therapy. The monitoring by blood tests that was carried out in the days following surgery were necessary to verify that a state of sepsis had not set in. There is not much data in the literature on the management of antibiotic coverage therapy required to avoid the risk of infections. Antibiotic prophylaxis to avoid an infectious pericarditis is certainly necessary, and thanks to clinical-laboratory monitoring it is possible to see if it is necessary to modify the therapy, also by means of possible blood cultures. In our case it was not necessary, as we noticed a rapid drop in all the inflammation indices in the days following the operation. On the other hand, as also described by Hiroaki M [7]. , in the case of purulent pericarditis with fever and laboratory signs of infection, targeted antibiotic and anti-inflammatory therapy must be initiated. The occurrence of pericardial effusion post-surgery may be a useful sign to evaluate for possible change of antibiotic therapy.

## Conclusions

The present report demonstrated successful closure of traumatic VSP via the right ventricular approach. Our case emphasizes the necessity of prompt surgery in the acute phase of traumatic VSP if medical treatment fails to stabilize circulatory dynamics.

## Data Availability

Not applicable.
